# Mandatory surveillance of bacteremia conducted by automated monitoring

**DOI:** 10.3389/fpubh.2024.1502739

**Published:** 2024-12-16

**Authors:** Kåre Mølbak, Christian Østergaard Andersen, Ram B. Dessau, Svend Ellermann-Eriksen, Sophie Gubbels, Thøger Gorm Jensen, Jenny Dahl Knudsen, Brian Kristensen, Lisbeth Lützen, John Coia, Bente Ruth Scharvik Olesen, Mette Pinholt, Flemming Scheutz, Ute Wolff Sönksen, Kirstine K. Søgaard, Marianne Voldstedlund

**Affiliations:** ^1^Epidemiological Infectious Disease Preparedness, Statens Serum Institut, Copenhagen, Denmark; ^2^Department of Veterinary and Animal Science, Faculty of Health, University of Copenhagen, Copenhagen, Denmark; ^3^Department of Diagnostic and Infectious Disease Preparedness, Statens Serum Institut, Copenhagen, Denmark; ^4^Department of Clinical Microbiology, Copenhagen University Hospital Amager and Hvidovre, Hvidovre, Denmark; ^5^Department of Data Integration and Analysis, Statens Serum Institut, Copenhagen, Denmark; ^6^Department of Clinical Microbiology, Zealand University Hospital, Slagelse, Denmark; ^7^Department of Regional Health Research, University of Southern Denmark, Odense, Denmark; ^8^Department of Clinical Microbiology, Aarhus University Hospital, Aarhus, Denmark; ^9^Department of Clinical Microbiology, Odense University Hospital, Odense, Denmark; ^10^Department of Clinical Microbiology, Rigshospitalet, Copenhagen, Denmark; ^11^Department of Infectious Diseases Epidemiology and Prevention, Statens Serum Institut, Copenhagen, Denmark; ^12^Department of Clinical Microbiology, Sygehus Lillebælt, Vejle, Denmark; ^13^Research Unit of Clinical Microbiology, Department of Regional Health Research, Esbjerg, Denmark; ^14^Department of Clinical Microbiology, Copenhagen University Hospital - Herlev and Gentofte, Herlev, Denmark; ^15^Department of Bacteria, Parasites and Fungi, The International Escherichia and Klebsiella Centre, Statens Serum Institut, Copenhagen, Denmark; ^16^Department of Bacteria, Parasites and Fungi, Statens Serum Institut, Copenhagen, Denmark; ^17^Department of Clinical Microbiology, Aalborg University Hospital, Aalborg, Denmark

**Keywords:** bacteremia, blood stream infection, surveillance, artificial intelligence, public health, AMR (antimicrobial resistance)

## Abstract

Except for a few countries, comprehensive all-cause surveillance for bacteremia is not part of mandatory routine public health surveillance. We argue that time has come to include automated surveillance for bacteremia in the national surveillance systems, and explore diverse approaches and challenges in establishing bacteremia monitoring. Assessed against proposed criteria, surveillance for bacteremia should be given high priority. This is based on severity, burden of illness, health gains obtained by improved treatment and prevention, risk of outbreaks (including health care associated infections), the emergence of antimicrobial drug resistance as well as the changing epidemiology of bacteremia which is seen along with an aging population and advances in medical care. The establishment of comprehensive surveillance for bacteremia was until recently conceived as an insurmountable task. With computerized systems in clinical microbiology, surveillance by real-time data capture has become achievable. This calls for re-addressing the question of including bacteremia among the conditions under mandatory surveillance. Experiences from several countries, including Denmark, show that this is feasible. We propose enhanced international collaboration, legislative action, and funding to address the challenges and opportunities.

## Introduction

Bacteremia is a major cause of morbidity and mortality worldwide (1, 2), but comprehensive all-cause monitoring is not included in mandatory routine public health surveillance with few exceptions (3–6). Rather, selected invasive infections including meningococcal diseases, pneumococcal disease, invasive *Haemophilus influenzae* infection, listeriosis and enteric fevers form part of national surveillance (7). Furthermore, selected drug-resistant bacteria including methicillin resistant *Staphylococcus aureus* (MRSA), vancomycin resistant enterococci (VRE) and carbapenemase producing enterobacterales (CPE) detected in blood cultures are targets for surveillance.

All-cause comprehensive and real-time bacteremia surveillance is relevant in its own right according to proposed criteria for surveillance (8) (Box 1). This is based on severity of bacteremia, health gains obtained by improving treatment and prevention, potential of the early investigation of outbreaks and other events requiring action, long-term burden of illness and dynamics of transmission (1, 2, 9–11). This is not a new idea; a national bacteremia registry in the United States was proposed in the 1960s but was never realized (12). Furthermore, it is rationally to operate the targeted surveillance systems, mentioned above, in one comprehensive system. This gives added value of data-extraction for existing surveillance and response to new developments in systemic infections.

BOX 1 **Concepts used in this paper**

Automated surveillance represents any form of surveillance where the **manual assessment and reporting are replaced by automated processes**. This includes fully automated and semiautomated data collection and case-ascertainment, validation, and analysis of denominator data [see also (41)].

Bloodstream infection is defined **by positive blood cultures in a patient with systemic signs of infection**. In automated surveillance, **bacteremia or fungaemia** will often serve as a proxy of bloodstream infection. Bloodstream infection will often have a focus. A discussion of the further relation to the clinical counterpart of sepsis is beyond the scope of the present paper.

Comprehensive surveillance includes **bacteremia or fungemia independent of the causative pathogen**, as well as place of onset (community or health care), health care association, or classification as primary or secondary bacteremia/fungemia. A comprehensive system should have a defined geographic catchment area.

The aim of this narrative review is to address whether time has come to include bacteremia in the national surveillance systems, and consider different approaches and challenges in establishing bacteremia surveillance. An accompanying paper by Dessau et al. (13) describes the epidemiology of bacteremia in Denmark and underpins the feasibility and utility of comprehensive bacteremia surveillance.

## Objectives of surveillance

Objectives of surveillance for bacteremia include outbreak detection, detection of emerging strains, the assessment of trends, analysis of risk factors, clinical outcomes and overall disease burden (10). The implementation of a comprehensive surveillance system would for example enable the early detection of new infections and allow for accurate tracking of changes in incidence and epidemiology. For instance, when a pathogen like *Neisseria meningitidis* is detected, healthcare professionals can implement infection prevention measures and post-exposure prophylaxis, providing significant public health benefits. Additionally, detection of emerging infections such as *Candida auris* (14), hypervirulent *Escherichia coli* and *Klebsiella pneumoniae* (15), or unusual pathogens, including *Aeromonas* and *Edwardsiella* spp. causing health care associated infections or foodborne outbreaks, would provide valuable data for clinical and public health interventions.

Community-onset bacteremia has a burden of illness that is similar to those of major trauma, acute stroke, and myocardial infarction (16). As mentioned by Laupland and Church, heart disease has garnered great attention and financial support by public and private organizations, but this is less the case for severe bacterial infections. Hence, a comprehensive surveillance for bacteremia will also serve as a tool to create awareness to gain support for enhanced efforts (17).

Furthermore, a comprehensive system will be important for setting priorities for policies, interventions and research. *Escherichia coli*, *Staphylococcus aureus*, and *Streptococcus pneumoniae* are often mentioned as frequent causes of community-onset bacteremia, and are in many settings responsible for more than one-half of the cases overall (18, 19). The incidence of fungal infections including systemic infections caused by *Candida* spp. has risen in the last decades, which calls for including fungemia in the development of the surveillance (14, 20). Current mandatory surveillance data do not reflect this overall development in the epidemiology of blood stream infections, and attempts to mitigate selected “superbugs” such as MRSA, VRE, and CPE may have little impact on the overall burden of these infections. Thus, comprehensive surveillance systems may be helpful in establishing appropriate political focus on the challenges, including the prevention of community-onset bacteremia and its clinical counterpart sepsis (21, 22). With the increasing use of whole genome sequencing (WGS), it becomes feasible to combine agreed procedures for typing with comprehensive epidemiological surveillance. The establishment of such a surveillance system will be a very powerful tool for public health and infection prevention, although its implementation has challenges (23).

Surveillance for bacteremia can also serve as a backbone for clinical databases and intervention activities and shall be seen in synergy with those. Furthermore, as other surveillance systems, data can serve as a source for the generation of hypotheses for research and development. A major objective is to serve as the backbone for antimicrobial drug resistance (AMR) surveillance which will be further explained below.

## The changing epidemiology of bacteremia

One of the important reasons for proposing surveillance is the changing epidemiology of bacteremia, including an increase in the incidence (in particular in patients >70 years of age) and the emergence of multi-drug resistant (MDR) pathogens (18, 24, 25). The reasons for this change may be related to several factors (Table 1). A surveillance system will be an important step toward a better understanding of the root causes of this change and may open new ways to intervene. Surveillance, with proper dissemination of information and guidance, will be a benefit for all clinical specialties, including oncology, hematology, nephrology, cardiology and surgery; specialties which see many of the patients at-risk of bacteremia. It has been reported that up to 30% of patients with bacteremia receive inappropriate empirical antibiotic therapy (18, 26–28) which has been associated with poor outcome (29, 30). With large differences in the prevalence of MDR pathogens across countries, timely data on the local distribution of the species and the patterns of AMR is thus pivotal to inform guidance to clinicians.

**Table 1:** Possible explanations for the increase in the incidence of bloodstream infections.

Factor	Explanation	References
Demographic changes including an aging population	Hospitals are facing a change in case-mix over time, including an increase of older adults patients who are at risk of bloodstream infections.	(37, 53)
Novel and more complex medical care	More invasive procedure, an increasing proportion of patients surviving treatment for malignant disease, and new patient groups receiving immunosuppressive treatment.	(54)
Increased rates of blood culturing and improved detection practices	Rising investigation density and better organization of emergency care increases the number of positive cultures. The use of automated incubation and detection system increases the efficacy of the laboratory.	(13, 17, 37, 55, 56)
The emergence of AMR resistant strains	Resistant strains causing additional infections rather than replacing those caused by susceptible bacteria.	(24)
Other factors including globalization	Importation of strains acquired abroad or from health facilities in other countries, changes in pathogen specific transmissibility or virulence, including global spread of microorganisms may add to the increased number of detections.	(57, 58)

## Surveillance for bacteremia as the backbone for AMR surveillance

In recent years, the threat of the emergence and spread of antimicrobial resistant bacteria is becoming urgent. European and international surveillance schemes have mainly targeted specific bacteria such as Methicillin-resistant *Staphylococcus aureus* (MRSA), ESBL producing enterobacterales, VRE or CPE. This focus leaves a number of questions. Do the drug-resistant bacteria affect the same patient risk-groups as their more sensitive counterparts, or are there risk groups that require special attention? What are the clinical consequences of infection with drug-resistant organisms compared with susceptible organisms? What is the excess burden of AMR organisms in terms of increasing incidence? How much of the increase can be attributed to increased awareness and detection practices, and do the increases follow that of other organisms causing bacteremia? These are examples of questions that are difficult to address without access to comprehensive data including denominator data, e.g., information on numbers of blood cultures taken, outcome hereof and culturing practices.

The availability of timely data on drug-resistance will potentially provide information of value for both clinicians and public health. Plasmid borne ampC-lactamase producing enterobacterales are important targets for AMR surveillance, but less recognized than other types of resistance mechanisms (31, 32). Another example is the categorization of carbapenemase-producing bacteria by molecular class, which would offer critical information for clinical as well as public health management (33). A timely monitoring of the spread of VRE would inform whether admission screening is necessary and whether switching from vancomycin to other drugs is advisable. Unbiased data on the incidence of infections with drug-resistant invasive pathogens would also facilitate analyses of the correlation between antimicrobial usage and the emergence of resistant bacteria.

Data from comprehensive monitoring will also be helpful in placing AMR in context. While much attention is on bacteremia caused by MRSA, the major part of burden of illness from *Staphylococcus aureus* may come from methicillin-sensitive strains (MSSA), in particular in the Northern European countries (34, 35). In the EU and EEA countries, the total number of bacteremia caused by *S. aureus* increased by 57% from 2005 to 2018. Notably, the number of MSSA increased by 84% whereas MRSA decreased by 31% (36). A narrow focus on prevention of MRSA seems of limited impact on the overall burden of disease due to *S. aureus* bacteremia, and this issue will be difficult to address unless surveillance is being redefined (35).

By obtaining a better picture of “the epidemiology of blood cultures,” clinicians and public health experts will be in a much better position to understand the drivers of emergence and spread of drug resistant organisms (37). However, bacteremia surveillance may also be of practical value in the support of interventions. As a part of an intervention effort to reduce the use of critically important antibiotics in Danish hospitals, we established an ongoing monitoring of 30 days case-fatality after blood was drawn for culturing. By withholding critically important antibiotics for the early empirical treatment of community acquired bacteremia, concerns were raised as regards reduced coverage of the drugs and potential worse outcomes among patients. Hence, a surveillance of case-fatality was therefore established in the form of a linkage between the Danish Microbiology Database (38) and the civil registry systems to monitor case-fatality. So far, the intervention has been implemented without an impact on fatality (39).

## Is it feasible to establish comprehensive bacteremia surveillance?

Surveillance for bacteremia has now become a manageable task in many countries or health care settings. Finland was the first country to monitor the epidemiology of bacteremia overall on a nationwide basis, and the surveillance is based on an electronic notification on positive blood cultures to the national surveillance institute (4, 40). England, Wales and Northern Ireland collect data from hospital microbiology laboratories, but this system is only mandatory for selected organisms (3). In parts of Canada, a novel surveillance system for blood stream infections was developed by linking health data from regional laboratories and hospital administrative data and using definitions for excluding contaminants and duplicate isolates (5). In Israel, almost all blood cultures, regardless of place of onset, are processed by laboratories in acute care hospitals that send monthly, mandatory reports of bacteremia to the National Center for Infection Control in the Ministry of Health (6). The PRAISE consortium, consisting of experts from public health institutes and hospitals in 10 European countries, has collected knowledge and experiences with large-scale implementation of automated surveillance, including bacteremia, and published a roadmap (41). In a spin-off project, work is being done to agree on minimal datasets and shared case definition for bacteremia across countries. Furthermore, ECDC has recently launched a project in order to support the transition of EU/EEA countries toward the most efficient use of routinely collected electronic health records for surveillance of infectious disease. The aim is to pilot and implement surveillance systems based on automatic extraction, cleaning, validation, and sharing of health data stored electronically in EU/EEA countries’ health information systems and other similar sources. The vision is to include reports from blood cultures in this system.

In Denmark, experience with automated surveillance in the Healthcare-Associated Infections Database (HAIBA) shows that it is possible to define a nationwide case definition for BSI and define whether it is healthcare-or community associated, and establish a fully automated production system and visualization for this (42). With the most recent revision of the legislation, a fully automated system has become part of the national surveillance scheme.

These examples show that comprehensive surveillance is within reach. Beyond the availability of electronic health records and a national civil registration system with unique national personal identification numbers, a number of other factors have contributed to the success in Denmark. This include the establishment of a national network of clinical microbiologists and epidemiologists, common protocols for data exchange between laboratories, and the development of common tools for mapping of codes (translating local codes to national codes) (38).

## Challenges and opportunities

A number of studies have validated electronic or automated surveillance systems, and it is generally agreed that automated systems are more efficient than conventional infection surveillance methods, including completeness, sensitivity and specificity (5, 42–46). Nonetheless, there are challenges to address.

Bacteremia is deemed to be present when a microorganism associated with disease is demonstrated from blood of an infected patient. It is therefore a requisite in any definition of bacteremia that a positive test, usually a blood culture, is present. However, a positive blood culture does not necessarily imply blood stream infection. Bacteremia or fungemia may arise from contamination, or a true positivity in the absence or presence of associated clinical diseases. In individual patient care, a clinician will determine the presence of an infection by an integration of available microbiological-, clinical-, immune response indicators and imaging techniques as well as the exclusion of contamination. Such an individual approach has significant limitations for surveillance purpose. Detection of microorganisms in blood cultures are common, and manual interpretation and reporting based on individual assessment will be time-consuming, is likely to be incomplete, and will lead to substantial interobserver variability (17). Therefore, electronic surveillance will rely on carefully developed algorithms for case definitions and for the exclusions of contaminants. New technologies such as mass spectrometry have contributed significantly to species identification and older systems for categorization based on phenotypic methods have become redundant. In the future, the increased application of molecular methods and use of artificial intelligence may open new ways of interpreting the raw data (47). It is important to establish international collaboration around the development and validation of these algorithms, including machine learning models to automate workflows and assist interpretation. This type of collaboration may also include other objectives, including sharing experiences from various countries’ surveillance systems in order to strengthen global response capabilities, and possibly the adoption of new antimicrobial agents that have not yet been approved.

A surveillance system optimally includes metadata such as patient age and gender, selected clinical characteristics, co-morbidity, management, health care association or community association, place of disease-onset and relevant risk factors. Linkage with hospital administrative data or clinical data can serve as the basis for deducting some of these characteristics (42, 48), whereas others may need to be collected in separate electronic or manual workflows. Hence, even a very advanced system may require some manual work to maintain high quality of the content (41). Further, the vision of national surveillance of bacteremia will not replace the need for high-quality clinical databases because the details of the data per case-patient will usually be limited in a surveillance system, whereas a clinical database contains detailed clinical information.

There are a number of practical and legal aspects (including GDPR) associated with data capture from existing databases including laboratory information management systems (which may have private or public ownership), patient administrative data, and electronic health records containing clinical information. Countries with national civil registries such as the Nordic countries have possibilities of linkage using a personal identification number (49), whereas other countries or health maintenance systems should use other approaches. Again, international exchange of national experiences is encouraged. Similarly, there may be practical and legal aspects in sharing data with users of the surveillance system to ensure that data reach those, who need to act. Establishing the necessary legal framework and securing funding for the implementation and operation of the surveillance system is crucial. Ensuring the system’s sustainability is of utmost importance.

When establishing comprehensive automated surveillance there is a need to evaluate skills and resources in the organization, as the implications may weigh heavier than in traditional surveillance. This includes data standardization, interoperability, data storage and-sharing, programming of algorithms, quality control, change and incident management (50). Effective governance structures are also crucial (51).

Collaboration is needed to leverage on artificial intelligence and machine learning to analyze patterns in the emergence of bacterial and fungal infections, provide resistance information, and facilitate characteristics that can help develop predictive models and algorithms for identifying region-specific infection patterns.

A final challenge relates to the contextualization of the available information. In traditional surveillance systems, patients are visible when nurses and doctors collect information “at the bedside.” However, IT systems and automation strategies can end up generating new forms of data work and fragmentation of clinically relevant information. Hence, a full surveillance system includes the capacity to recontextualize and visualize the data, i.e., “to tell the stories behind the numbers” in an actionable way (52). This is not a straightforward task and requires commitment not only by the team responsible for the surveillance system but also the health organization and the policy makers.

## Conclusion

Due to a number of determinants, including an aging population, shifts in health care, improved treatment of chronic and malignant diseases, and globalization, the epidemiology of both community and health care associated bacteremia and fungemia is changing. These changes have been documented in research, but the dissemination of the findings has been slow, uncoordinated and not sufficiently supported by a legal and organizational framework that can lead to action. Although AMR has been identified as a major threat to health, surveillance remains primarily targeted toward selected pathogens and certain resistance mechanisms and “drug-bug combinations,” and is not supported by comprehensive data on detection practices and disease incidence. Furthermore, the current systems are rarely suitable toward the early detection of new emergences.

The ongoing technological reforms in health care systems, including the use of electronic databases in clinical microbiology and molecular methods for detection and identification, open new possibilities for establishing mandatory comprehensive surveillance systems for bacteremia and fungemia. Experiences from several countries show that this is a feasible task. It will be beyond the scope of this review to suggest a roadmap for this process, but it can be divided in layers, including governance, taking advance of the existing information-and communication technology, data analytics, integration of genomic sequencing, access to metadata and data storage, and dissemination [see also (41)]. Further, financial implications and manpower requirements are important. With this background, we propose enhanced international collaboration, legislative action, and funding to address the remaining opportunities and challenges.
